# Cell biomechanics and mechanobiology in bacteria: Challenges and opportunities

**DOI:** 10.1063/1.5135585

**Published:** 2020-04-01

**Authors:** Christine E. Harper, Christopher J. Hernandez

**Affiliations:** 1 Meinig School of Biomedical Engineering, Cornell University, Ithaca, New York 14853, USA; 2 Sibley School of Mechanical and Aerospace Engineering, Cornell University, Ithaca, New York 14853, USA

## Abstract

Physical forces play a profound role in the survival and function of all known forms of life. Advances in cell biomechanics and mechanobiology have provided key insights into the physiology of eukaryotic organisms, but much less is known about the roles of physical forces in bacterial physiology. This review is an introduction to bacterial mechanics intended for persons familiar with cells and biomechanics in mammalian cells. Bacteria play a major role in human health, either as pathogens or as beneficial commensal organisms within the microbiome. Although bacteria have long been known to be sensitive to their mechanical environment, understanding the effects of physical forces on bacterial physiology has been limited by their small size (∼1 *μ*m). However, advancements in micro- and nano-scale technologies over the past few years have increasingly made it possible to rigorously examine the mechanical stress and strain within individual bacteria. Here, we review the methods currently used to examine bacteria from a mechanical perspective, including the subcellular structures in bacteria and how they differ from those in mammalian cells, as well as micro- and nanomechanical approaches to studying bacteria, and studies showing the effects of physical forces on bacterial physiology. Recent findings indicate a large range in mechanical properties of bacteria and show that physical forces can have a profound effect on bacterial survival, growth, biofilm formation, and resistance to toxins and antibiotics. Advances in the field of bacterial biomechanics have the potential to lead to novel antibacterial strategies, biotechnology approaches, and applications in synthetic biology.

## I. INTRODUCTION

In the classic text *On Growth and Form*, the mathematical biologist D'Arcy Thompson described the role of physical forces in the development and growth of organisms, including examples from mammals, arthropods, and individual eukaryotic cells.^1^ Physical forces regulate development, healing processes in organs throughout the body, and the pathogenesis of diseases.^2–4^ Although biomechanics and mechanobiology are well recognized in mammals and model organisms used in medical research, eukaryotes represent only a small portion of the diversity and abundance of life on Earth. The total biomass of bacteria and archaea is ∼40 times greater than that of all animals combined (and 1300 times more biomass than humans).^5^

Bacteria are ubiquitous in the environment and exhibit a broad influence on many areas including human health (as both pathogens and as beneficial commensals), the health of ecosystems, all aspects of the food chain, biofouling of devices, processes used in molecular biology and biotechnology, and emerging technologies such as synthetic biology. Although commonly viewed as colloidal particles in suspension (as bacteria are observed suspended in media in laboratory conditions), bacteria, in nature, are more commonly found adhered to surfaces or to each other in biofilm communities. Bacteria can survive challenging mechanical environments including the fluid shear stresses generated by turbulent flows, extreme hydrostatic pressures in soils and deep oceans, and interruptions in structural integrity caused by antibiotics and other toxins.^6^ The ability of bacteria to not only resist mechanical loads (biomechanics) but also to respond to changes in the mechanical environment (mechanobiology) is necessary for their survival.

The idea that bacteria are sensitive to physical forces is not new. In 1982, Koch and colleagues proposed that growth and elongation of individual bacteria were related to mechanical stress and strain within the cell envelope associated with turgor pressure.^7^ Mechanosensitive channels in the bacterial cell membrane were subsequently identified and shown to promote survival in response to rapid fluctuations in osmolarity.^8,9^ Technological advances in the past decade have related the mechanical properties of bacteria to cell division^10^ and cell envelope remodeling^11,12^ and have shown that mechanical stimulation of bacteria influences key pathogenic processes including expression of virulence factors^13^ and biofilm formation.^14^

Here, we review the current state of the art of the emerging field of bacterial biomechanics and mechanobiology. Although, in the past decades, there have been considerable advances in understanding bacterial locomotion^15^ and the mechanics of biofilms as an aggregate,^16,17^ the current manuscript focuses on recent advances in understanding the biomechanics and mechanobiology of the bacterial cell body and non-locomotory organs and cell envelope components. The review is intended as an introduction to persons familiar with cell and molecular biomechanics in mammalian cells.

### A. Structure of bacteria: Differences from mammalian cells

The biomechanics and mechanobiology of bacteria differ considerably from those of mammalian cells, primarily due to differences in cell physiology and structure. The structure, morphology, and internal constituents of bacteria vary considerably among species. However, there are three major characteristics that make bacteria biomechanically distinct from mammalian cells: the bacterial cell envelope, the magnitude of internal pressure, and the cytoskeleton.

**FIG. 1.**
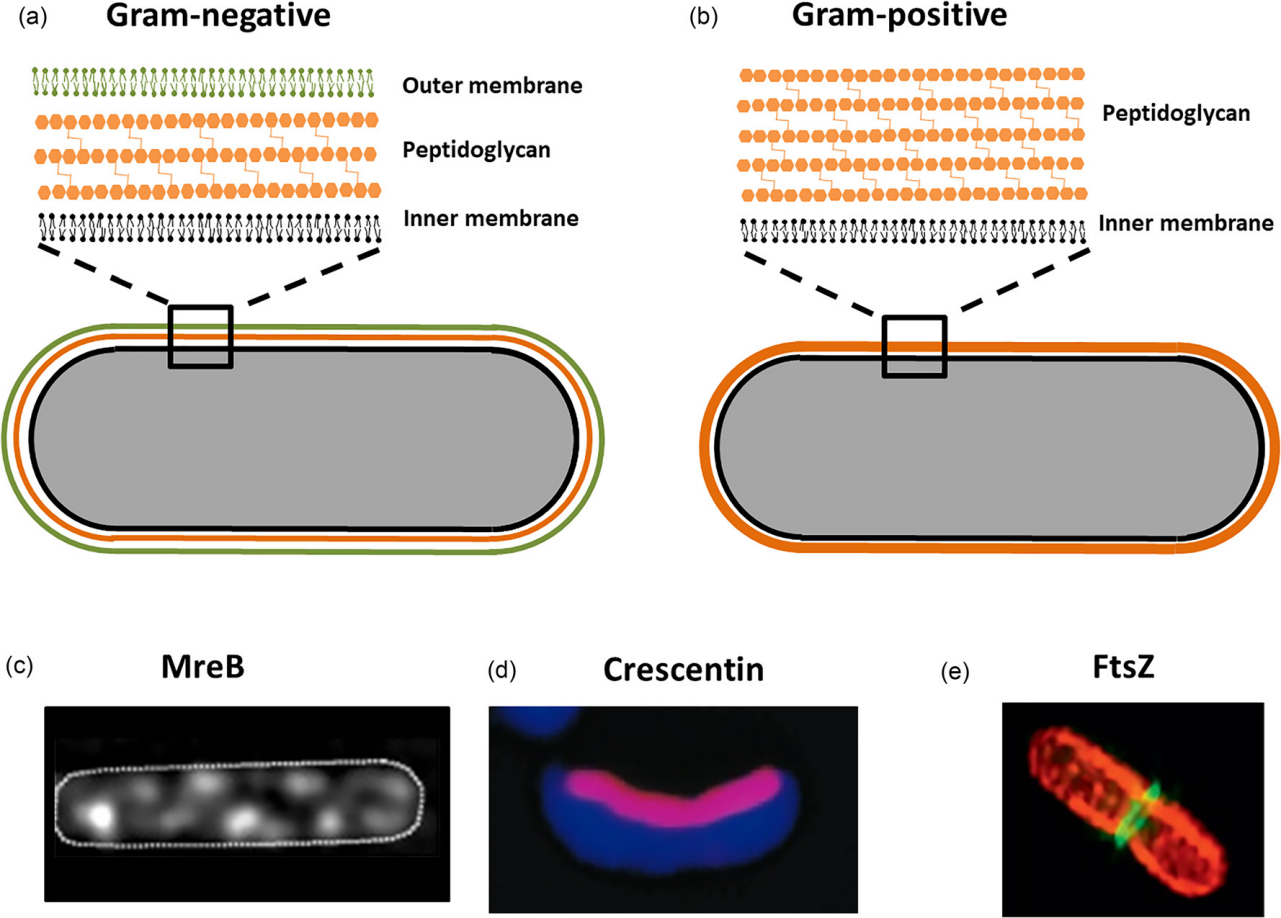
(a) Gram-negative bacteria have an inner membrane, peptidoglycan, and an outer membrane. (b) Gram-positive bacteria have an inner membrane and peptidoglycan. (c) MreB, shown in white, is located circumferentially in rod-like bacteria. Reprinted with permission from Domínguez-Escobar *et al.*, Science **333**(6039), 225–228. Copyright 2011 The American Association for the Advancement of Science. (d) Crescentin filaments, shown in pink, are oriented on the inner surface of the cell wall in curved and helical bacteria. Reprinted with permission from Ausmees *et al.*, Cell **115**(6), 705–713. Copyright 2003 Elsevier. (e) FtsZ, shown in lime green, assembles at the septum during cell division. Reprinted with permission from Cohen *et al.*, Methods Enzymol. **551**, 211–221. Copyright 2015 Elsevier.

Mammalian cells are separated from their environment by a cell membrane consisting of a phospholipid bilayer. Structurally, the cell boundary of bacteria is more complex than that of mammalian cells and can include one or two cell membranes and, in most bacterial species, a cell wall. Together the cell membrane(s) and cell wall are referred to as the “cell envelope.” The constituents of the cell envelope provide the primary phenotypic classification for bacteria as determined using staining approaches discovered by Hans Christian Gram:^18^ Gram-negative bacteria have an inner membrane, an outer membrane, and a cell wall [Fig. 1(a)] while Gram-positive bacteria have only the inner membrane and cell wall [Fig. 1(b)]. The inner membrane and outer membrane of bacteria, like that of mammalian cells, are composed of phospholipid bilayers. The lipid compositions of bacterial cell membranes and mammalian cell membranes are different in many ways. For example, the most abundant lipid in mammalian cells, phosphatidylcholine, is not present in most bacteria.^19,20^ In mammalian cells, cholesterol is a vital component of the plasma membrane, but cholesterol is not found in bacterial cell membranes. The differences in the membrane composition between mammalian and bacterial cells lead to differences in membrane fluidity. Additionally, the protein composition within bacterial membranes differs from that within mammalian membranes. Most notably, the outer membrane of Gram-negative bacteria contains lipopolysaccharides (LPS) that contribute to the outer membrane structure and may also influence cell mechanical properties. Finally, the bacterial cell wall is the major determinant of bacterial cell shape and mechanical properties. Mammalian cells do not have cell walls. The bacterial cell wall is composed of peptidoglycan (known as murein in older literature^21^). Peptidoglycan consists of polysaccharide strands crosslinked by peptide chains, creating a porous, mesh-like structure (pore size 4–20 nm).^22,23^ The thickness of the cell wall varies among species; the peptidoglycan layer in Gram-positive bacteria is reported to be 19–33 nm thick and the peptidoglycan of Gram-negative bacteria is reported to be 2.5–6.5 nm thick.^24^ As a result, Gram-positive bacteria display greater cell envelope stiffness than Gram-positive bacteria.^25^

In mammalian cells, transmembrane protein complexes that transmit and/or detect mechanical forces are involved in most mechanosensory mechanisms. In bacteria, mechanically analogous protein complexes must cross the entire cell envelope. Bacterial trans-envelope protein complexes that connect all layers of the bacterial cell envelope and are present in both Gram-positive and Gram-negative species include the flagella and pili.^25^ In Gram-positive organisms, wall teichoic acids are attached to the peptidoglycan and lipoteichoic acids form connections between the inner membrane and the peptidoglycan. In Gram-negative organisms, lipoprotein (LPP) connects the outer membrane and peptidoglycan, and a variety of trans-envelope protein complexes, including secretion systems and efflux complexes, span the inner membrane, cell wall, and outer membrane.

Mammalian cells survive in a relatively well controlled osmotic environment, and in most cases, the cell membrane needs only resist small magnitudes of osmotic pressure; internal pressure in mammalian cells is typically less than one kilopascal.^26^ In contrast, under normal physiological conditions, bacteria maintain a large trans-envelope osmotic pressure, referred to in the microbiology literature as turgor pressure. Turgor pressure is typically 100 kPa,^27^ although reports range from 10 to 500 kPa.^28–31^ The large range in reported turgor pressure may be a result of differences among bacterial genus and species and is also likely the result of technical limitations in measuring turgor pressure in bacteria.^32^ In either case, the turgor pressure in bacteria is at least one order of magnitude greater than that of mammalian cells (and likely two orders of magnitude greater). As a result, membrane tension in bacterial cells is large, creating mechanical challenges to cells undergoing cell envelope remodeling, cell elongation, and cell division.

In mammalian cells, the mechanical performance of cells is dominated by the cytoskeleton—the dynamic structure of actin and tubulin that provides structure to the cell and enables transfer of forces to internal structures and organelles. Although bacteria lack a cytoskeleton, bacteria do have a number of cytoskeletal-like molecules that serve mechanical functions. The most well understood cytoskeleton-like proteins are MreB, crescentin, and FtsZ. MreB was first identified as a “shape determining” molecule in *Bacillus subtilis*. MreB and MreB analogs are present in almost all non-spherical bacteria.^33^ MreB is a curved protein located circumferentially in rod-like bacteria and resists the hoop stresses generated by turgor pressure [Fig. 1(c)].^34–36^ In the absence of MreB, a normally rod shaped species assumes a spherical shape.^37^ Crescentin is a molecule that resembles intermediate filaments in mammalian cells and is present in crescent and helical shaped bacteria.^38^ Crescentin filaments are oriented longitudinally along the inner surface of the bacterial cell envelope, creating asymmetric mechanical stiffness that results in the cell curvature as the cell elongates during growth [Fig. 1(d)].^38–40^ FtsZ is a tubulin-like molecule that assembles at the point of cell division^41^ (known as the Z-ring) [Fig. 1(e)]. FtsZ resists circumferential and hoop stresses caused by turgor pressure and thereby enables the tapering of the cell envelope required for cell separation during division.^42–44^

### B. Mechanical stimulation assays

The mechanical properties of bacteria and bacterial components are challenging to evaluate due to the small cell size (typically ∼1 *μ*m in characteristic size). The small size of bacteria represents a challenge in applying mechanical loads as well as measuring deflections of the cell envelope. As a result, many methods for measuring mechanical properties of mammalian cells or applying mechanical stimulus to mammalian cells are not feasible for bacteria. Substrate stretching, one of the most common methods of mechanical stimulation of mammalian cells, is ineffective for bacteria because it requires bacteria to be adhered to the substrate in at least two points (a situation difficult to confirm experimentally). Additionally, deformation of a bacterium on a substrate can be difficult to observe. Micropillar substrates, another useful means of measuring forces generated by mammalian cells, are also ineffective for bacteria as the size of the micropillars (2–10 *μ*m) is substantially larger than that of bacteria.^45–47^

Micropipette aspiration is a well-established approach for measuring the stiffness of the mammalian cell membrane but has limited utility with bacteria. During micropipette aspiration, a pipette tip much smaller than the cell applies negative pressure to the cell membrane to pull a small portion of the cell into the pipette tip. The pressure and deformation of the cell are measured and used to calculate elastic modulus and viscosity of the cell.^48^ Although nanopipettes can be made that are sufficiently small to capture a bacteria, the stiffness of the cell prevents reliable measures of deformation under micropipette aspiration (although some measures of bacteria after the removal of the cell wall have been reported^49^).

One early and innovative method of measuring bacterial mechanical properties was created by Thwaites and Mendelson. Bacteria grown into chains were formed into threads up to a meter long, and the material properties of these threads were used to estimate the material properties of individual bacteria.^50^ A major limitation of examining threads of bacteria is that the measured stiffness is related not only to the stiffness of the bacteria but also to the proteins linking the bacteria that are loaded in series within the thread.

Despite the challenges in providing a controlled mechanical stimulus to bacteria, a number of techniques have been developed to mechanically stimulate bacteria to assess cell mechanical properties or even isolate mechanosensitive mechanisms.

#### 1. Osmotic shock

**FIG. 2.**
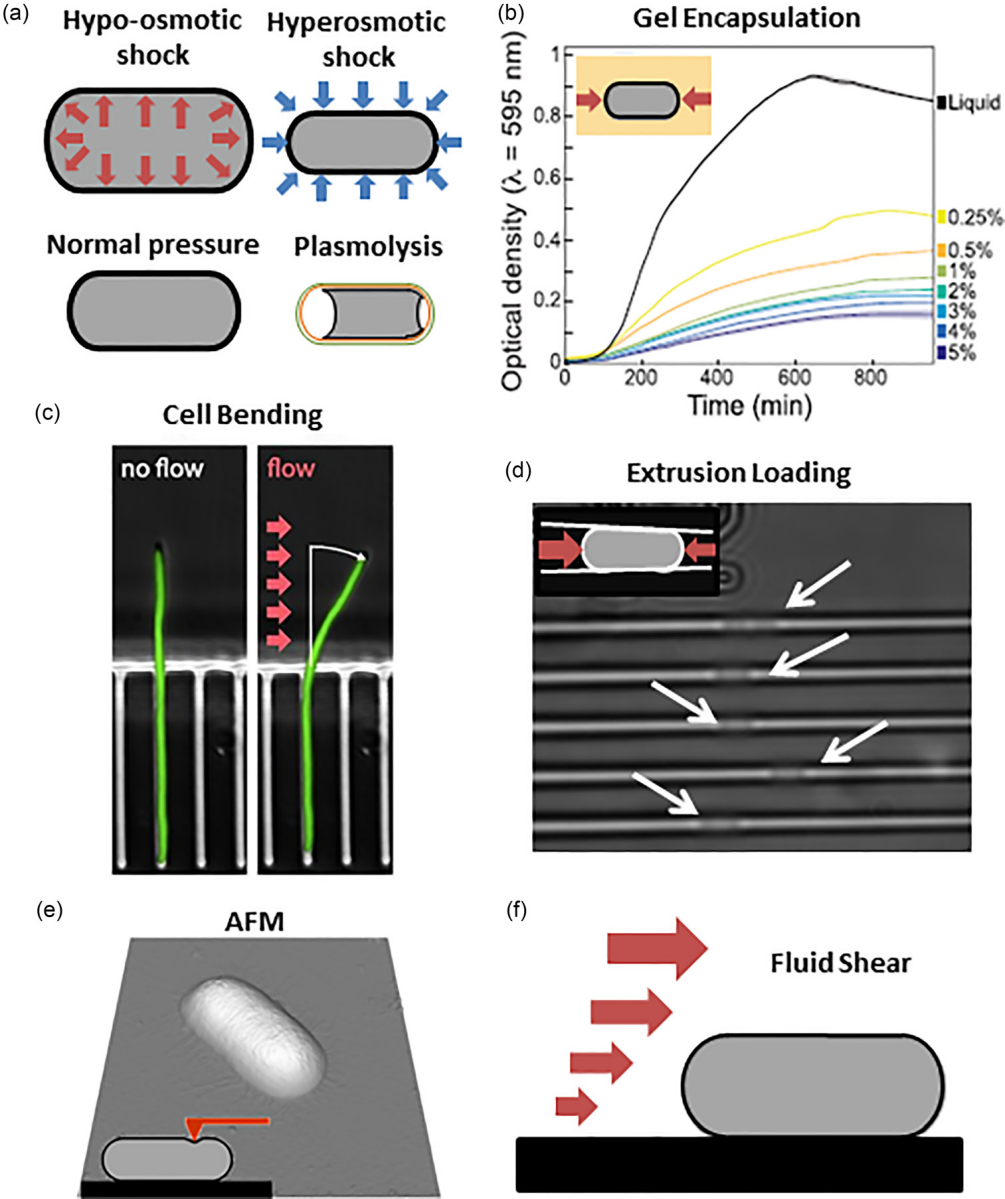
Existing methods for mechanically stimulating bacteria. (a) Osmotic shock. Top left: a bacterium experiences hypo-osmotic shock which results in an increase in cell volume. Top right: a bacterium experiences hyper-osmotic shock which results in a decrease in cell volume. Bottom left: a bacterium under normal osmotic pressure. Bottom right: a bacterium experiences plasmolysis which may result in the inner membrane (black) separating from the cell wall (orange). (b) Gel encapsulation. In gel encapsulation, the bacterium experiences compressive axial forces. The optical density of a gel with encapsulated cells over time is dependent upon gel stiffness. Reprinted with permission from Auer *et al.*, Cell Syst. **2**(6), 402–411. Copyright 2016 Elsevier. (c) Cell bending. Fluid flow on free end of the filamentous bacterium causes cell bending. Reprinted with permission from Amir *et al.*, Proc. Natl. Acad. Sci. U. S. A. **111**(16), 5778–5783 (2014). Copyright 2014 National Academy of Science of the United States of America. (d) Extrusion loading. Bacteria are forced into tapered channels using microfluidic pressure. (e) Atomic force microscopy. A small region of a cell is displaced by a cantilever during AFM. A height profile of a bacterium created using AFM. From Domingues *et al.*, *Atomic Force Microscopy*. Copyright 2019 Springer Nature. Reprinted with permission from Springer Nature. (f) Fluid shear. Fluid flow over a surface-attached bacterium.

Perhaps the most straightforward method of applying mechanical stress to bacteria is through osmotic shock. The technique involves generating a rapid change in the osmolarity of the external media, resulting in changes in turgor pressure and bacterial geometry [Fig. 2(a)]. Changes in cell dimensions during osmotic shock can be used to estimate cell envelope stiffness.^51,52^

During hypo-osmotic shock, the osmolarity outside of the cell drops quickly, resulting in an increase in turgor pressure, cell volume, and tension on the bacterial cell envelope as water diffuses into the cell. The change in mechanical stress within the cell envelope during osmotic shock is determined using mechanical models of thin walled pressure vessels.^53,54^ For a rod-like cell with internal pressure P, radius r, and wall thickness t, the thin walled pressure vessel approximation indicates the following principal stresses:

$$\begin{array}{c} \sigma_{a}=\frac{Pr}{2t},\\ \sigma_{h}=\frac{Pr}{t},\\ \sigma_{r}=-\frac{P}{2}, \end{array}$$ (1)

where σ_a_ is the axial stress, σ_h_ is the hoop stress, and σ_r_ is the radial stress (much smaller in magnitude than the other two and denoted as negative because it is compressive in nature). During hypo-osmotic shock, the magnitude of axial stress, hoop stress, and radial stress in the cell envelope increases (Table I).

**TABLE I.** The change in the magnitude of the mechanical stress sin the bacterial cell envelope caused by each of the mechanical loading approaches described in the text is shown.

	Hoop stress	Radial stress	Axial stress
Osmotic shock			
Hypo-osmotic	↑	↑	↑
Hyperosmotic	↓	↓	↓
Gel encapsulation	…	…	↓
Cell bending	…	…	↑ Flow side
			↓ Opposite of flow
Extrusion loading	↓	↓	↑
AFM	↑ near contact	↓ at contact	↑ near contact
Fluid shear	…	…	↑

Hyper-osmotic shock can be caused by a rapid increase in osmolality of the surrounding liquid media and results in reductions in turgor pressure, cell volume, and stresses within the cell envelope. If the reduction in turgor pressure is sufficiently large, the inner membrane may separate from the cell wall, a response known as plasmolysis [Fig. 2(a)]. Observations of plasmolysis have been useful for determining the mechanical contributions of the different components of the cell envelope.^52^ However, separation of the inner membrane from the cell wall through plasmolysis is rarely uniform around the cell envelope, and it is therefore difficult to estimate the resulting change in membrane tension.

Osmotic shock is advantageous in that it can be used on bacteria of any shape and size, and changes in cell geometry due to fluctuations in osmolarity can be observed quickly, on the order of seconds to minutes. Microfluidic devices developed over the last decade and available commercially can apply rapid changes in osmolarity including cyclic variations, opening up a range of potential mechanical stimuli to bacteria. Additionally, osmotic shock can be performed in a high-throughput fashion. Osmotic shock experiments have provided substantial insight into the role of pressure, cell wall insertion, and the stiffness of the outer membrane.^11,52,55^ Despite the utility of osmotic shock as a mechanical loading approach, there are limitations to the amount of mechanical loading that can be applied using this method. When large changes in mechanical load are applied, hyper-osmotic media may lead to plasmolysis and hypo-osmotic media may lead to cell lysis. Additionally, modifications to osmolarity, by altering cell water content, may influence cell physiology independently of the change in turgor pressure, potentially limiting the ability to use the approach for studying mechanobiology.^55^ Finally, a major limitation of osmotic shock as a mechanical stimulus is that the magnitude of turgor pressure is difficult to ascertain (see above), limiting our ability to understand the relative changes in mechanical stress.

#### 2. Gel encapsulation

Tuson *et al.* introduced a novel method of studying bacterial mechanical properties/mechanobiology by encapsulating bacteria within solid media, a technique we refer to here as gel encapsulation.^54^ In a gel encapsulation experiment, rod-shaped bacteria are suspended in solid media (agarose gel) and the elongation of each individual bacterium is measured as it grows. The gel surrounding the bacteria generates compressive forces that resist elongation [Fig. 2(b)]. Increasing the stiffness of the gel results in greater resistance to cell elongation. By examining cell elongation in many different stiffnesses of gel, it is possible to calculate the Young's modulus of the bacterial cell envelope.^54^

Mechanical stress caused by gel encapsulation involves compressive forces generated by the gel that balance the tensile stresses in the cell envelope associated with turgor pressure. As a result, a rod-shaped bacterium submitted to gel encapsulation experiences a reduction in axial tensile stress, without changes in hoop or radial stress (Table I).^56^ One challenge in interpreting mechanical characterization using gel encapsulation is that the forces generated by the surrounding gel only occur when the gel is deformed by the addition of the material to the cell envelope. As there is evidence that external forces can influence the rates of insertion of material into the cell envelope, the factor being measured (rate of elongation) influences the applied force, complicating interpretation of the results.^54^

However, gel encapsulation has a major advantage in that it can be used in a high throughput fashion. Auer and colleagues found that optical density measures of bacteria within gels of different stiffness were useful surrogate measures of cell lengthening [Fig. 2(b)].^57^ By measuring optical density measurements in 96 well plates loaded with different stiffness gels, Auer and colleagues were able to estimate the relative stiffness of the entire Keio collection of *Escherichia coli* (a library of *E. coli* strains in which each strain has one non-essential gene knocked out) and thereby identify genes associated with cell stiffness.^57^ Similarly, a library of *Pseudomonas aeruginosa* was recently screened to identify genes associated with cell stiffness.^58^ However, there are limitations to the gel encapsulation method. So far, this method has only been used on rod-shaped bacteria. Furthermore, if the cells are close together within the gel, the growth of one cell may affect the compressive force of the gel on nearby cells.

#### 3. Cell bending

Cantilever bending is a common approach for measuring the mechanical properties of a material. There are two methods for performing a cantilever bending test with live bacteria: one using a microfluidic flow based device^59^ and another using an optical trap probe.^36^ In the microfluidic flow approach, a bacterium within a microchannel has cell division inhibited to induce filamentous growth. The cell grows outside of the microchannel into a chamber where a transverse fluid flow is present that bends the cell [Fig. 2(c)]. The optical trap approach involves binding one end of the bacterium to a substrate and the other end to a polylysine-coated microsphere. An optical trap is then used to apply force to the polylysine-coated bead causing the free end of the cell to bend. The displacement of the tip of the cell during bending is used to determine cell flexural rigidity using beam theory.^59^ The Young's modulus of the cell envelope is then determined from the flexural rigidity by assuming a cell envelope thickness.

An advantage of the bending approaches is the ability to apply a controlled force that can be maintained from seconds to hours. This approach is also unique in its ability to directly measure flexural rigidity. Although mechanical analysis under bending is straightforward, there are a number of limitations to this approach. First, the requirement that cell division is inhibited (i.e., the cells become filamentous) results in changes in cell physiology that may affect mechanical properties and mechanosensory mechanisms.^60^ Second, the cells cannot be recovered from the testing apparatus to allow biochemical assays. Furthermore, with regard to the optical trap method, it is difficult to image the contact between the cell and the underlying substrate, resulting in some errors in the shape and location of the boundary conditions with the underlying surface. This limitation with the optical trap method may explain why the approach has not yet been used since its initial presentation. Finally, both of these approaches are labor-intensive and therefore have relatively low throughput.

#### 4. Extrusion loading

Extrusion loading is a mechanical stimulation approach resembling micropipette aspiration used in mammalian cells.^48^ Rather than pulling the cell into a micro-/nano-pipette, the approach involves forcing the cell into a small tapered channel. The first reported implementation of the concept was performed within a microfluidic device.^61^ Bacteria flow into the wide end of the tapered channel and get lodged in the channel [Fig. 2(d)]. The distance the bacterium travels down the tapered channel is related to the stiffness of the bacteria as well as the applied hydrostatic fluid pressure. Bacteria submitted to extrusion loading remain viable and continue to elongate and divide while experiencing mechanical load.

Extrusion loading generates a complex mechanical stress state within the bacteria.^56^ A bacterium trapped within the channel decreases in diameter as it deforms within the tapered channel. The contact between the walls of the tapered channel results in a radial compressive force applied to the trunk of the bacterium that acts against hoop stresses generated by turgor pressure (Table I). The contact with the channel wall also results in a small frictional force along the axial direction. A bacterium submitted to extrusion loading increases in length axially and experiences an increase in axial tensile stress.

During extrusion loading, the hydrostatic fluid pressure at the inlet of the microfluidic device is 25–200 kPa.^56,61^ Hydrostatic pressure considered alone, in the absence of the extrusion loading microfluidic device, causes changes to the stress state within bacteria. Extreme magnitudes of hydrostatic pressure (>50 MPa) have been shown to cause changes in bacterial physiology and cell death.^62^ At hydrostatic pressure from 50 to 100 MPa, cell growth, DNA replication, and RNA synthesis inhibition have been observed.^62^ Very high hydrostatic pressures, 100–600 MPa, are commonly used in the food industry to inactivate or kill bacteria in order to preserve or sterilize food.^63^ However, these extreme hydrostatic pressures are at least an order of magnitude greater than the hydrostatic pressure used during extrusion loading.

An advantage of extrusion loading is that it can be used on hundreds of bacteria at up to 12 distinct load magnitudes at once. Additionally, the approach does not require alterations in bacterial cell physiology (inducing filamentation, forcing adhesion to a surface) and can theoretically be used on bacteria with any initial shape (rod-like, crescent, cocci, etc.). Current limitations to the approach include the complicated stress state of the cell envelope as compared to other approaches. Additionally, as with any microfluidic based approach, removing bacteria from the device for additional biochemical examination has not been performed and would be technically challenging.

#### 5. Atomic force microscopy

Atomic Force Microscopy (AFM) has also been used by a number of groups to query the mechanical properties of bacteria. AFM has been used to measure the stiffness of the cell envelope of the isolated bacterial cell wall,^64^ to measure the stiffness of the cell envelope in an intact cell,^29,64–66^ to determine the viscoelastic properties of the cell envelope,^67,68^ and to estimate turgor pressure.^29,31,65,66^ AFM can also be used to created profiles of a bacterium.^69^

The AFM probe comes into contact with the bacteria and displaces a small region of the cell surrounding the probe [Fig. 2(e)]. Detailed analytical and/or finite element models are used to assess the mechanical stresses within the cell envelope when in contact with the AFM probe. Directly beneath the point of contact, the cell envelope experiences compression, but the surrounding regions experience tensile stresses.

Advantages of AFM include being able to query local regions of interest within a bacterium and being able to apply very precise load magnitudes. One limitation of using AFM is that the cells must be immobilized in order to probe them mechanically. Immobilization can be achieved in many ways, including entrapment in pores, electrostatic interactions, and polyethyleneimine or gelatin coated slides.^66,67,70^ An additional limitation is that the boundary conditions related to cell contact with the substrate and initial turgor pressure can be difficult to control but can greatly influence the results of mechanical models used to interpret the results of the AFM study. Additionally, AFM measurements are labor-intensive resulting in low throughput. Finally, the AFM approach has been shown useful for probing cell biomechanics but has not yet been shown useful for studying mechanobiology (the response to mechanical stimuli).

#### 6. Fluid shear

Fluid shear is an additional method for applying mechanical stimuli to bacteria. Bacteria naturally adhere to surfaces (the first step in biofilm formation).^71^ When an adhered bacterium is submitted to additional fluid flow, stresses are generated within the cell envelope^72^ [Fig. 2(f)]. Fluid shear experiments have provided insight into the response of bacteria to mechanical stimuli. In *P. aeruginosa*, fluid shear enabled the identification of an operon that responds to the flow rate independent of shear stress.^73^

The specific mechanical stresses generated by fluid flow are complicated. Bacteria adhere to surfaces using adhesins and other exposed surface proteins.^72^ Depending on the concentration of adhesion points, there are stress concentrations within the cell envelope around the points of adhesion. Additionally, fluid flow over the cell causes shear stress in the cell envelope. Further stress can develop if the bacterium begins to move across the surface using pili or flagella. An advantage of this method is that it closely mimics mechanical stresses observed in the environment. A limitation of this approach is that the complicated boundary conditions associated with adhesion points make it challenging to evaluate cell biomechanics.

## II. MECHANOBIOLOGY IN BACTERIA

In addition to being useful for assessing the mechanical properties of bacteria, the approaches described above have the potential to help understand how bacteria respond to environmental mechanical stimuli. Here, we will review some major findings in bacterial mechanobiology regarding membrane protein function, cell wall insertion, surface sensing, fluid flow sensing, and biofilm properties. We refer the reader to recent reviews focusing entirely on bacterial mechanosensing for more information about molecular mechanisms.^74,75^

Some of the earliest evidence that bacteria respond to mechanical stimuli came from the discovery of mechanosensitive channels in *E. coli.*^76,77^ Hypo-osmotic shock causes a sudden increase in bacterial turgor pressure and cell envelope stress, and as a result, stretch-activated mechanosensitive channels open and release solutes. The release of solutes through the mechanosensitive channels results in a decrease in the osmotic gradient and turgor pressure, thereby helping prevent cell lysis.^78^ Mechanosensitive channels are now recognized in many Gram-positive and Gram-negative species.^79,80^

Mechanical stresses within the bacterial cell envelope are directly experienced by trans-envelope protein complexes. One important function of trans-envelope protein complexes is removal of antibiotics and other toxins. In a recent study, Genova *et al.* found that the CusCBA efflux complex (used by bacteria to remove toxic copper and silver) is disrupted in response to mechanical stresses in the cell envelope.^56^ Disruption of the CusCBA efflux complex was correlated with deviatoric stresses within the cell envelope, but not with cell envelope hydrostatic stress. This finding is novel in linking one form of mechanical stress (deviatoric stress) within the cell envelope to a biological response and suggests that mechanical stresses have the potential to influence a broad array of toxin and antibiotic resistance mechanisms in bacteria.

Mechanical stress and strain have been shown to influence the localization of cell wall insertion in rod-shaped bacteria. Ursell and colleagues monitored the localization of new cell wall insertion in filamentous *E. coli.* Filamentous *E. coli* display curvature, dimpling, and bulging. New cell wall insertion occurred preferentially at areas of negative cell wall curvature,^81^ a mechanism that eventually leads to straightening of the cell. Although the authors argued that the localization of new cell envelope insertion was dominated by curvature, regions of negative cell wall curvature also correspond to stress concentrations and it is unclear if curvature or mechanical stress is the dominant cause. More recently, Wong and colleagues used the microfluidic cell bending approach to show that insertion of the new cell envelope material occurred preferentially in regions of the cell experiencing tensile strain.^82^ The coupling of mechanical stress and strain with cell wall insertion may contribute to maintenance of a stable geometry.

Mechanical forces have also been implicated in bacterial surface sensing and surface adhesion. Surface adhesion is essential to colonization and biofilm formation, although it is still not clear how bacteria sense a surface in order to initiate adhesion.^71,83^ The mechanical environment at the surface differs from the mechanical environment in a bulk liquid; a cell physically deforms with contact to a surface, shear stress increases as a cell transitions from a flowing fluid to a surface. Additionally, mechanical forces associated with adhesion are often transmitted through extracellular appendages physically interacting with the surface. These and other mechanical cues could help initiate surface adhesion. Type IV pili, flagella, and some cell envelope proteins may have mechanosensitive functions that contribute to surface sensing.

Type IV pili are extracellular appendages that dynamically extend and retract from bacteria and are important for attachment and twitching motility on surfaces. In *P. aeruginosa*, the attachment of Type IV pili to a solid surface begins a signaling cascade that regulates hundreds of genes.^84^ The mechanical forces caused by surface attachment lead to the release of virulence factors in *P. aeruginosa* only when the Type IV pili are functional.^13^ Additionally, in surface attached *P. aeruginosa*, fluid shear leads to increases in cyclic dimeric guanosine monophosphate (cyclic di-GMP), a factor that is essential in initiating biofilm formation, only when the Type IV pili are functional.^14^

Flagella are well known for their role in bacterial locomotion, but flagella are also important for regulating surface adhesion.^85^ Bacteria may sense a surface through inhibition of flagellar rotation. As a bacterium nears a surface, rotation of the flagellum is interrupted by contact with the surface resulting in stresses within the motor complex. External inhibition of flagellar rotation may influence many surface behaviors including biofilm formation, adhesion, and swarming. Restriction of flagellar rotation in *B. subtilis* upregulates the DegS–DegU pathway, which promotes biofilm formation.^86^ Restriction of flagellar rotation in *Caulobacter crescentus* leads to the production of polar holdfast adhesive polysaccharide that helps with surface adhesion.^87^ In *Vibrio parahaemolyticus* restriction of the polar flagella promotes differentiation to the swarmer cell phenotype, motility on surfaces.^88,89^ In addition to sensitivity to inhibition, flagella assembly appears to be sensitive to the magnitude of mechanical load. Flagellar motor units assemble and disassemble in response to changes in external load.^90–93^ External load on flagella can be changed by adjusting media viscosity, attaching beads of varying sizes to flagellar filaments, and tethering cells to a surface using flagellar stubs. More detailed information on flagellar mechanosensing can be found in a review by Belas.^94^

The Cpx-signaling pathway potentially serves a mechanosensitive function during surface adhesion. The Cpx-signaling pathway is activated due to a variety of stimuli including cell attachment to hydrophobic surfaces and response to misfolded proteins in the cell envelope.^95^ CpxA is an inner membrane protein that phosphorylates CpxR, a cytoplasmic response regulator that activates transcription.^95,96^ Mutations to the CpxA gene result in poor adhesion and biofilm formation.^97^ NlpE is an outer membrane lipoprotein that is essential for activation of the Cpx pathway during surface adhesion; however, NlpE is not essential for other Cpx responses, such as response to misfolded proteins in the cell envelope.^95^ Together this suggests that NlpE, CpxA, and CpxR may sense and respond to the mechanical forces the cell envelope experiences during surface contact.

Once cells are adhered to a surface, the forces generated by surrounding fluid shear can lead to changes in virulence factors and biofilm formation. In enterohemorrhagic *E. coli*, increased fluid shear enhances the expression of a group of virulence genes encoded in the locus of enterocyte effacement (LEE).^98^ In *P. aeruginosa* attached to a surface, fluid shear leads to increases in cyclic-di-GMP, a factor that is essential in initiating biofilm formation.^14^ Furthermore, a recent study indicated that, in *P. aeruginosa*, an operon may respond to flow rate independently of fluid shear stress.^73^

Mechanical forces due to fluid flow have been implicated in altering the mechanical properties and matrix composition of bacterial biofilms. Biofilms grown at greater magnitudes of fluid shear tend to be more rigid than biofilms grown at lower magnitudes of fluid shear.^99–101^ The changes in biofilm mechanical properties may be explained by changes in extracellular polysaccharide density. Greater magnitudes of fluid shear stress during biofilm production have been shown to lead to increased polysaccharide production in *Pseudomonas fluorescens*,^102^ *Staphylococcus aureus*,^103^ and *Bacillus cereus.*^104^ Changes in biofilm properties related to growth at greater fluid shear may make biofilms more viable in environments with transient increases in shear stress. In *B. cereus*, biofilms grown under greater magnitudes of shear stress were more capable of remaining adhered to the surface when exposed to increased shear stress.^104^ Similarly, *P. aeruginosa* biofilms grown at greater magnitudes of shear stress were more strongly adhered and more cohesive.^105^ There is some evidence that biofilm properties in clinical infections are influenced by the mechanical environment. Clinical isolates of *Staphylococcus epidermidis* were collected from areas of high shear, such as catheters, and areas of low shear, such as cerebral spinal fluid shunts. Cells from high shear areas were more likely to produce biofilms mediated by polysaccharide intercellular adhesin.^106^ Although these studies indicate that fluid shear can influence the biofilm composition/structure, how forces are transduced remains to be determined.

## III. CONCLUSIONS AND OUTLOOK

Bacterial physiology is important to human health, agriculture, biofouling of devices, molecular biology and biotechnology, and food production and security. Studies to date suggest that bacteria vary widely in terms of mechanical properties and that mechanical stress and strain influence key physiologic mechanisms in bacteria including cell division, biofilm formation, and resistance to toxins and antibiotics. Although understanding the role of physical forces in bacterial physiology has the potential to advance a number of fields, there are major technical limitations to further discovery. A major limitation to further advances in studying bacterial biomechanics and mechanobiology is that there is only one high-throughput method of stimulating bacteria, and that method (gel encapsulation) provides limited control over the applied mechanical load. A high throughput methodology with more rigorous control over the mechanical stresses on the bacteria could enable more broad use of genetic screens and selection to identify biomechanical and/or mechanobiologic phenotypes and mechanisms. Existing methods of applying mechanical forces to bacteria either provide precision in biomechanical assessment or the ability for further biochemical examination, but not both, which presents a limitation to understanding mechanotransduction mechanisms. Finally, there have been relatively few studies that have isolated the mechanical contributions of individual structures and proteins within bacteria. Although classically viewed as relatively homogeneous fluid structures, recent advances in microscopy have revealed much more detailed internal structures within bacteria that each may play a role in the response to mechanical stimuli. Improved understanding of the role of mechanical forces in bacteria physiology has the potential to lead to new engineering and synthetic biology applications in which the mechanical function of bacteria is a key component.
